# Insights into the unique characteristics of hepatitis C virus genotype 3 revealed by development of a robust sub-genomic DBN3a replicon

**DOI:** 10.1099/jgv.0.001486

**Published:** 2020-09-08

**Authors:** Joseph C. Ward, Sebastian Bowyer, Shucheng Chen, Guilherme Rodrigues Fernandes Campos, Santseharay Ramirez, Jens Bukh, Mark Harris

**Affiliations:** ^1^​ School of Molecular and Cellular Biology, Faculty of Biological Sciences and Astbury Centre for Structural Molecular Biology, University of Leeds, Leeds, LS2 9JT, UK; ^2^​ Copenhagen Hepatitis C Program (CO-HEP), Department of Infectious Diseases, Hvidovre Hospital, Kettegård Allé 30, DK-2650 Hvidovre, Denmark; ^3^​ Department of Immunology and Microbiology, Faculty of Health and Medical Sciences, University of Copenhagen, Blegdamsvej 3, DK-2200 Copenhagen N, Denmark; ^†^​ Present address: São Paulo State University, Institute of Biosciences, Languages and Exact Sciences, Cristóvão Colombo Street, 2265, Post Code 15054-000, São José do Rio Preto, São Paulo State, Brazil

**Keywords:** hepatitis C virus, sub-genomic replicon, genotype 3, NS5A

## Abstract

Hepatitis C virus (HCV) is an important human pathogen causing 400 000 chronic liver disease-related deaths annually. Until recently, the majority of laboratory-based investigations into the biology of HCV have focused on the genotype 2 isolate, JFH-1, involving replicons and infectious cell culture systems. However, genotype 2 is one of eight major genotypes of HCV and there is great sequence variation among these genotypes (>30 % nucleotide divergence). In this regard, genotype 3 is the second most common genotype and accounts for 30 % of global HCV cases. Further, genotype 3 is associated with both high levels of inherent resistance to direct-acting antiviral (DAA) therapy, and a more rapid progression to chronic liver diseases. Neither of these two attributes are fully understood, thus robust genotype 3 culture systems to unravel viral replication are required. Here we describe the generation of robust genotype 3 sub-genomic replicons (SGRs) based on the adapted HCV NS3-NS5B replicase from the DBN3a cell culture infectious clone. Such infectious cell culture-adaptive mutations could potentially promote the development of robust SGRs for other HCV strains and genotypes. The novel genotype 3 SGRs have been used both transiently and to establish stable SGR-harbouring cell lines. We show that these resources can be used to investigate aspects of genotype 3 biology, including NS5A function and DAA resistance. They will be useful tools for these studies, circumventing the need to work under the biosafety level 3 (BSL3) containment required in many countries.

## Introduction

Hepatitis C virus (HCV) infects 70 million individuals worldwide. In 85 % of the 2 million annual new cases, infection leads to chronic hepatitis, eventually causing liver diseases such as fibrosis, cirrhosis and hepatocellular carcinoma (HCC). HCV has a positive-strand RNA genome and is the most variable human virus, classified into eight genotypes (GTs) and exhibiting >30 % nucleotide sequence divergence, with each GT being further divided into subtypes [1–3]. GT3 is the second most common GT (after GT1) and accounts for 30 % of global HCV cases [4, 5]. The burden of GT3 infection falls disproportionately on low-to-middle income countries (LMICs); in particular, 70 % of HCV infections in Pakistan, India and Thailand are GT3 [6], and it is believed that the global dissemination of GT3 is in part due to population migration from these countries. Consistent with this, GT3 is highly prevalent in the UK (47 % of HCV cases), and in other parts of Western Europe.

HCV GT3 is of interest as it presents higher levels of resistance to the direct-acting antivirals (DAAs) that are now extensively used to treat HCV infection; in particular, GT3 isolates are more resistant to the class of DAAs that target NS5A [e.g. the first in class drug daclatasvir (DCV)] [7]. In addition, compared to other GTs, GT3 infection is associated with a higher incidence of insulin resistance and steatosis (fatty liver), leading to a more rapid progression to chronic liver disease [4]. The reasons underpinning these two unique characteristics remain to be fully elucidated. In this context, the study of GT3 biology would be greatly facilitated by the development of robust *in vitro* replication systems, both infectious clones and sub-genomic replicons (SGRs). To this end, two groups established GT3a SGRs from serum-derived full-length cDNA clones (S310 and S52) [8, 9]. In both cases. passage of G418-resistant colonies resulted in the acquisition of cell culture-adaptive mutations that enhanced replication. Despite these mutations, replication rates for these SGRs remained low compared to the ‘gold standard’ GT2a JFH-1-derived SGRs [10], perhaps reflecting the lack of *in vitro* infectivity of the full-length clones used for their creation [8, 11].

A significant breakthrough was the development of the DBN3a cell culture infectious clone (DBN3a_cc_) – the introduction of 17 substitutions across the genome resulted in levels of infectivity that were comparable to those for JFH-1 [12]. Here we describe the further development of SGRs derived from DBN3a_cc_ and their potential use for the study of GT3 DAA resistance and virus–host interactions.

## Methods

### Cell culture

Huh7.5 cells were cultured in Dulbecco’s modified Eagle’s medium (Sigma) supplemented with 10 % (v/v) foetal bovine serum (FBS), 100 IU ml^−1^ penicillin, 100 µg ml^−1^ streptomycin and 1 % (v/v) non-essential amino acids (Lonza) in a humidified incubator at 37 °C with 5 % CO_2_. An Huh7.5 cell line harbouring the SGR-neo-DBN3a replicon was generated by electroporation with *in vitro* RNA transcripts of SGR-neo-DBN3a. At 48 h post-electroporation (p.e.) cells were selected with G418 (700 µg ml^−1^) until cell colonies were visible. Colonies were then expanded and maintained in the presence of G418 at 400 µg ml^−1^.

### Generation of SGR constructs

An SGR-DBN3a construct without reporter was created using a synthetic gene string (Genewiz) covering the region from an unique *Nru*I site within the 5′ UTR to an unique *BsiW*I site within NS3 of DBN3a_cc_. This gene string encoded a truncated core region to permit functional IRES translation followed by two new unique restriction sites, *SnaB*I and *Mlu*I, and an EMCV IRES to drive the translation of the downstream HCV polyprotein NS3-NS5B. The *SnaB*I and *Mlu*I sites were used to introduce a firefly luciferase gene engineered to contain reduced CpG and UpA dinucleotide frequencies [13], neomycin phosphotransferase or eGFP reporters.

The SGR-eGFP-JFH-1 construct was made by the removal of the luciferase reporter gene from the SGR-luc-JFH-1 plasmid [10] using flanking *Pme*I and *Bgl*II restriction sites. The eGFP reporter was then generated by PCR amplification incorporating *Pme*I and *Bgl*II sites.

#### *In vitro* RNA transcription

SGR constructs were transcribed *in vitro* using 1 µg of linearized and purified DNA template and the RiboMax T7 Transcription kit following manufacturer’s protocol (Promega). Transcribed RNA was purified using RNA Clean and Concentrator-25 (Zymogen).

### Luciferase assays

Huh7.5 cells were used to study replicon replication. Cells were washed twice in ice cold phosphate-buffered saline (PBS) before electroporation of 4×10^6^ cells in PBS with 2 µg of RNA as described previously [14]. Cells were resuspended in complete media and seeded onto 96-well plates at 3×10^4^ cells/well. At 4, 24, 48 and 72 h p.e. cells were harvested by lysis with 50 µl of passive lysis buffer (Promega). Luciferase activity was determined on a BMG plate reader by the addition of 50 µl of luciferase assay reagent (Promega) to 50 µl of lysate.

### DAA assays

Sensitivity to DAAs was ascertained by electroporation of SGR-luc-DBN3a or SGR-luc-JFH-1 (original or Y93H mutants) into Huh7.5 cells as described above. At 4 h p.e. media were changed and replaced with complete media containing daclatasvir (DCV), cyclosporin A (CsA), velpatasvir (VEL) or sofosbuvir (SOF) in a titrating concentration as indicated. Electroporated cells were incubated for 72 h before lysis and luciferase activity was determined as described above.

### GFP replicon assays

Huh7.5 cells were seeded onto 24 well plates at 1×10^4^ cells per well. RNA transcripts of SGR-eGFP-DBN3a or SGR-eGFP-JFH-1 were prepared and 1 µg of RNA was transfected per well using Lipofectamine 2000 following the manufacturer’s protocol (Thermo Fisher). Four hours post-transfection, the medium was removed and replaced with complete medium before incubation in an IncuCyte ZOOM.

### Western blotting

Huh7 cells harbouring SGR-feo-JFH-1 or Huh7.5 cells harbouring SGR-neo-DBN3a were lysed using Glasgow lysis buffer (1 % Triton X-100, 120 mM KCl, 30 mM NaCl, 5 mM MgCl2, 10 % glycerol (v/v) and 10 mM PIPES, pH 7.2) with protease inhibitors and phosphatase inhibitors added. Proteins were separated by electrophoresis on a 7.5 % SDS-PAGE gel, and following electrophoresis, proteins were transferred onto PVDF membrane and blocked with 50 % (v/v) Odyssey blocking buffer (LiCor) in Tris-buffered saline (TBS). The membrane was then incubated with primary antibody as labelled, sheep anti-NS5A [15], rabbit anti-pS225 [16] or rabbit anti-pS232 [17] at 4 °C overnight. After washing with TBS, membranes were incubated with fluorescently labelled anti-sheep (800 nm) and anti-rabbit (680 nm) secondary antibodies for 1 h at room temperature. Unbound secondary antibody was removed, and membrane imaged on a LiCor Odyssey Sa fluorescent imager.

### Immunofluorescent microscopy

Huh7.5 cells harbouring SGR-neo-DBN3a were seeded at 1×10^5^ cells per well onto a 24-well plate. Cells were left to adhere before fixation using 4 % paraformaldehyde in dH_2_0 and permeabilized with 0.1 % (v/v) Triton X-100 (Sigma) for 7 min. Coverslips were washed twice in PBS and primary sheep anti-NS5A was applied in 10 % (v/v) FBS in PBS and incubated for 2 h at room temperature. Unbound primary antibody was removed before the addition of donkey anti-sheep Alexa Fluor 594 nm. Secondary antibody was incubated in the dark for 1 h and excess was removed by washing with PBS. Cells were then imaged in the Incucyte ZOOM.

For confocal microscopy cells were seeded onto glass coverslips and left to adhere for 24 h. Cells were fixed and stained with primary antibody as above. Unbound primary antibody was removed before the addition of donkey anti-sheep Alexa Fluor 594 nm. Secondary antibody was incubated in the dark for 1 h and excess was removed by washing with PBS. Coverslips were mounted on a microscope slide with ProLong gold antifade (Invitrogen). Confocal microscopy images were acquired using a Zeiss LSM 880 microscope.

## Results

### DBN3a-derived SGRs replicate efficiently in Huh7.5 cells

**Fig. 1.**
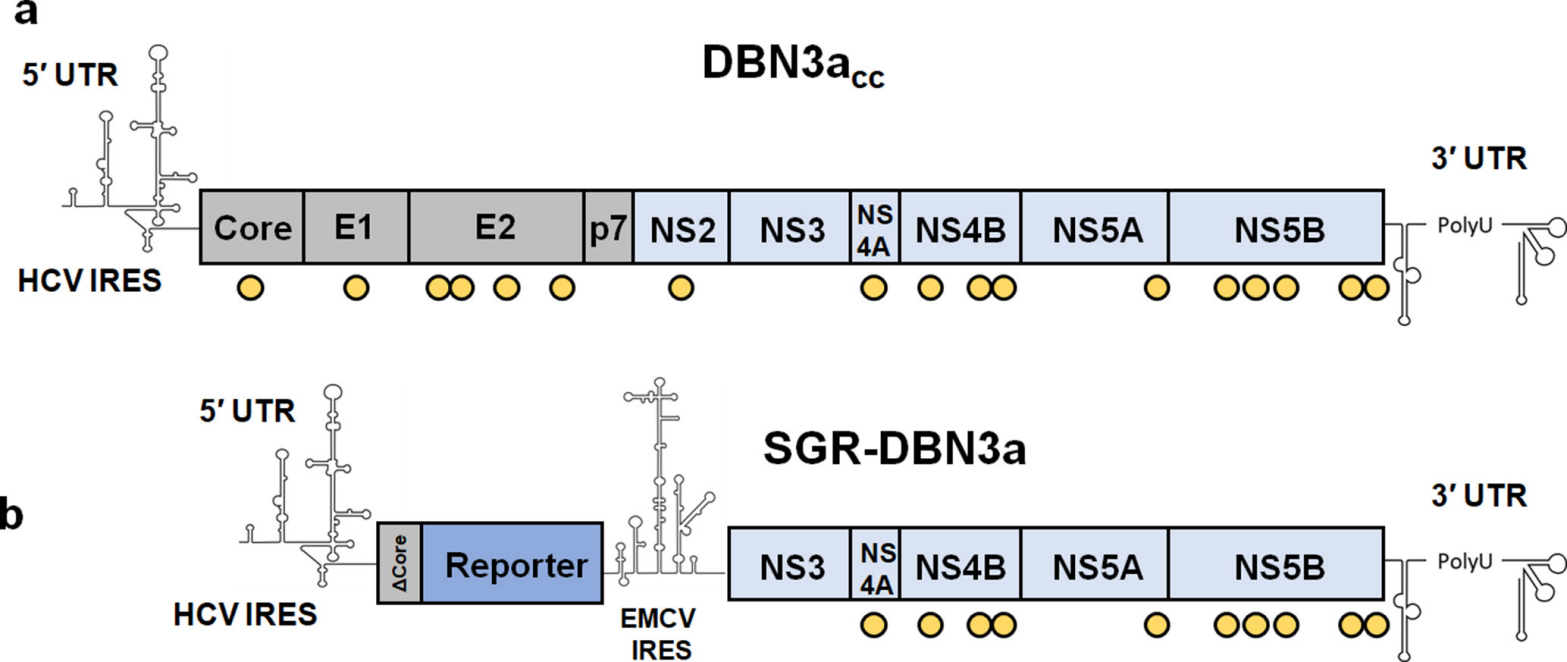
Structure of the DBN3a infectious clone and bicistronic SGRs. (a) Genome of the DBN3a_cc_ infectious clone with yellow circles indicating sites of mutation to enhance virus replication and propagation [12]. (b) Schematic of the DBN3a SGR: core, E1, E2, p7 and NS2 coding sequences have been removed, leaving only the 5′ end of the core sequence, coding for the N-terminal 12 amino acids. (ΔCore). The reporter (CpG/UpA-low firefly luciferase, neomycin phosphotransferase or eGFP) is thus expressed as a fusion with the N-terminus of core and is under the translational control of the HCV IRES. The HCV replicase, NS3-NS5B with DBN3a_cc_ adaptive mutations, is under the translational control of the EMCV IRES.

To provide a broad range of potential applications we generated HCV SGRs containing a panel of different reporters: a firefly luciferase gene engineered to contain reduced CpG and UpA dinucleotide frequencies [13], neomycin phosphotransferase and eGFP. These reporters were fused at the N-terminus to the first 12 residues of the DBN3a core protein (ΔCore) to ensure that full IRES activity was retained. Expression of the DBN3a NS3-NS5B proteins with infectious cell culture-adaptive substitutions was driven by a second IRES derived from EMCV (Fig. 1). As a negative control we generated a mutant in which the active site of the NS5B RdRp was mutated from GDD to GNN.

**Fig. 2.**
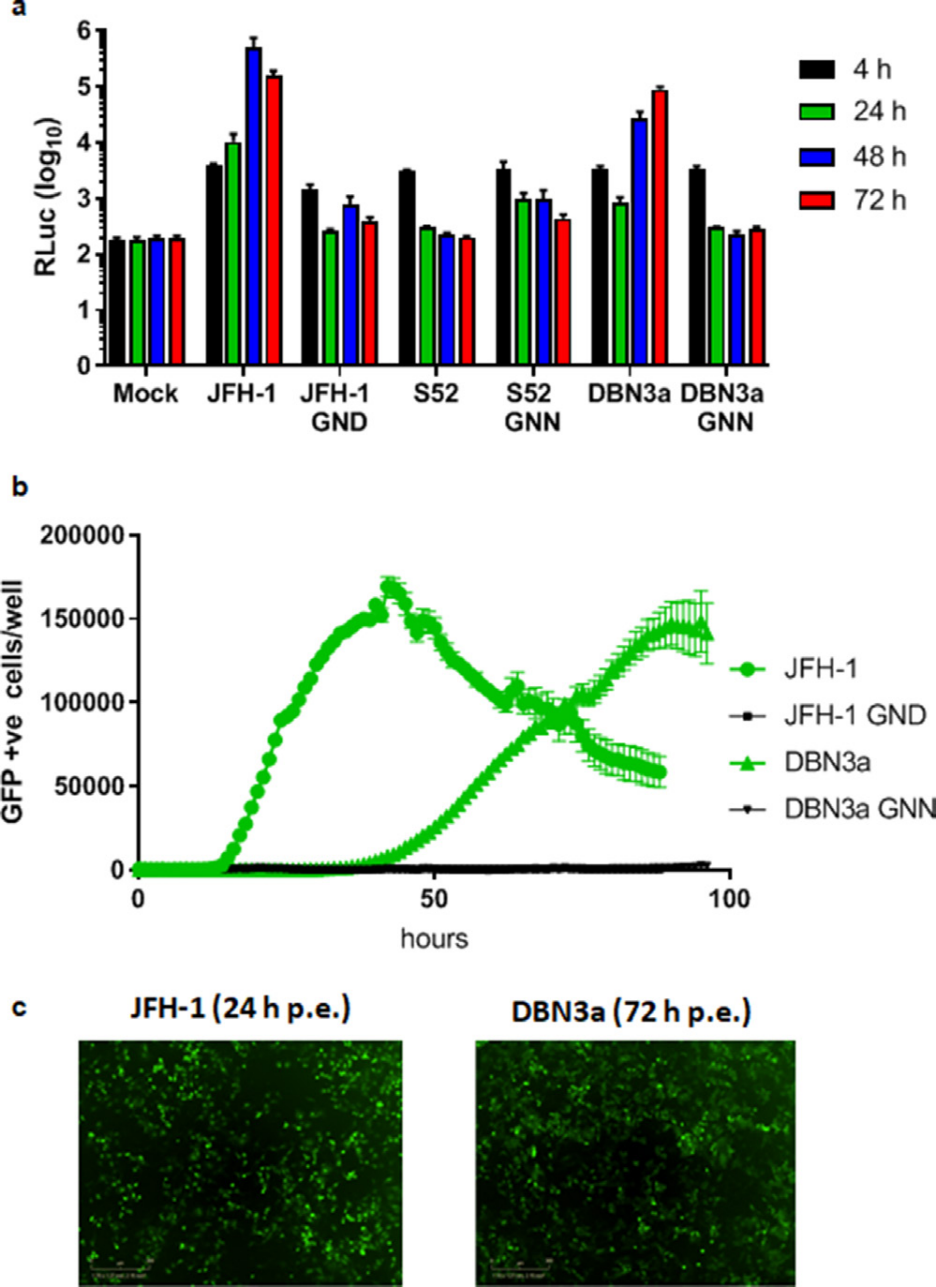
Transient replication of DBN3a-derived SGRs. (a) Huh7.5 cells were electroporated with *in vitro* transcripts of JFH-1 [10], S52 [9, 18] and DBN3a SGRs containing a CpG/UpA low luciferase, and replication was monitored by measuring the production of luciferase at 4, 24, 48 and 72 h p.e. GND/GNN: polymerase-inactive negative controls. *n*=3, error bars represent sem. (b) JFH-1 and DBN3a SGRs containing an eGFP reporter were transfected into Huh7.5 cells with corresponding GND/GNN-negative controls. Replication was monitored hourly for 96 h by measuring eGFP expression using an IncuCyte Zoom. *n*=2, error bars represent sem. (c) Images of JFH-1 and DBN3a eGFP SGR-transfected cells at 24 or 72 h p.e., respectively.

We first evaluated the transient replication of the SGR-luc-DBN3a in Huh7.5 cells and compared it to two other SGRs. Firstly, the GT3a isolate S52 (AII), which contained three culture-adaptive mutations (T1056A, T1429I and S2204I) [9, 18], referred to as S52 hereafter. Secondly, the GT2a isolate JFH-1 [10], which did not contain any culture-adaptive mutations. Polymerase-inactive (GNN) mutants of each of these SGRs were included in the assay. As shown in Fig. 2a, SGR-luc-JFH-1 replicated robustly, as expected, exhibiting a >100-fold increase in luciferase expression over 48 h, whereas replication of SGR-luc-S52 was almost undetectable, as observed previously [18]. Reassuringly, SGR-luc-DBN3a replicated efficiently, although it exhibited a delay compared to JFH-1, with reduced luciferase expression at 24 h compared to the increase seen in JFH-1. However, DBN3a did show a subsequent increase in expression at 48 and 72 h, reaching an approximately 100-fold increase in luciferase expression.

Using an SGR containing an eGFP reporter (SGR-eGFP-DBN3a), we were able to derive real-time kinetic data for the rate of replication of DBN3a using an IncuCyte ZOOM, an automated fluorescent microscope situated within an incubator (Fig. 2b). This approach has been used extensively for analysis of FMDV replication [19]. Using this real-time imaging, replication was assessed by the number of eGFP-positive cells, and we observed a similar delay in SGR-eGFP-DBN3a replication compared to SGR-eGFP-JFH-1. The latter showed detectable replication from 12 h p.e., increasing to a peak at 48 h. In contrast, no eGFP-positive cells were detected for SGR-eGFP-DBN3a until approx. 30 h p.e., peaking at 90 h, and declining thereafter. As expected, the polymerase-inactive mutants failed to accumulate eGFP positivity. Representative fluorescence images of the electroporated cells confirmed the similar levels of eGFP fluorescence at either 24 h p.e. for JFH-1 and 72 h p.e. for DBN3a (Fig. 2c). The sensitivity of the IncuCyte ZOOM was not sufficient to detect input translation from either JFH-1 or DBN3a constructs.

### Evaluation of NS5A expression and phosphorylation

**Fig. 3.**
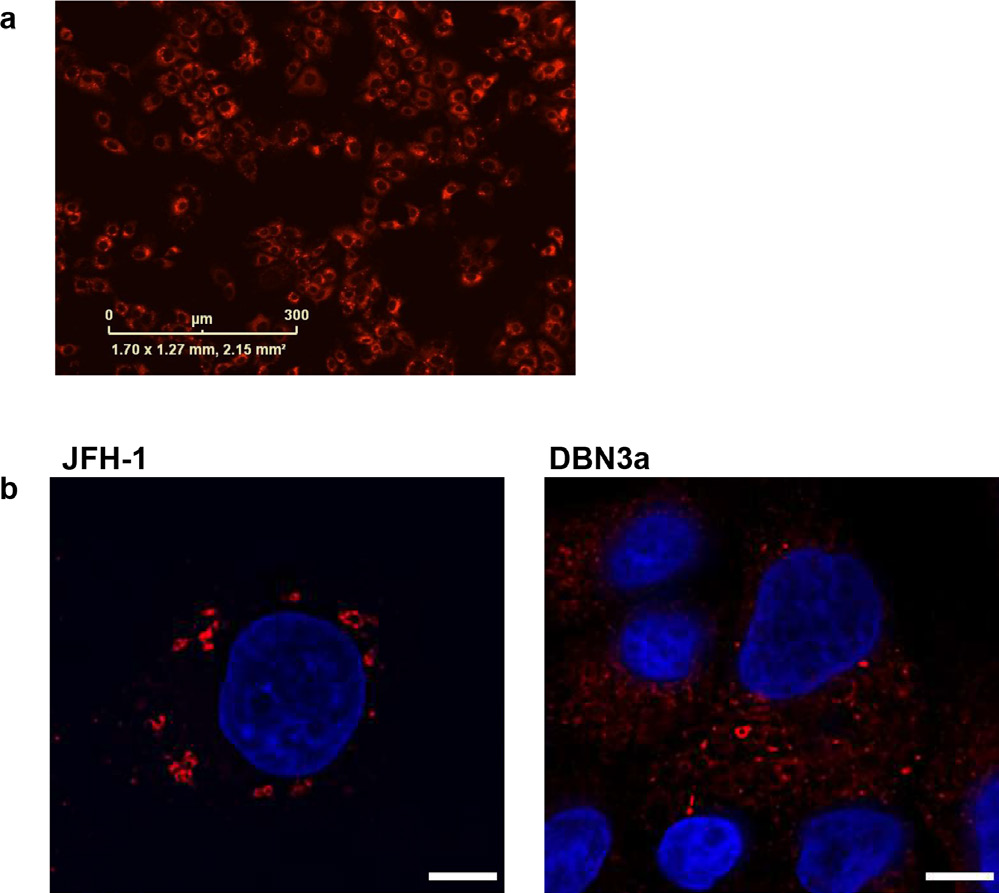
Sub-cellular localization of JFH-1 and DBN3a NS5A. (a) Huh7.5 cells were electroporated with SGR-neo-DBN3a RNA. SGR-harbouring cells were selected using G418. Cells were stained with an antibody to NS5A and visualized using immunofluorescent microscopy. (b) SGR-feo-JFH-1- or SGR-neo-DBN3a-harbouring cells were stained with an antibody to NS5A and visualized with confocal microscopy.

We also established a stable Huh7.5 cell line harbouring SGR-neo-DBN3a by selection with G418. As expected, immunofluorescence with an anti-NS5A antiserum revealed extensive cytoplasmic staining in the selected cell population (Fig. 3a). Confocal immunofluorescence microscopy was used to compare the distribution of NS5A with that observed in cells harbouring SGR-feo-JFH-1 [20] (Fig. 3b). In both cell populations NS5A exhibited punctate distribution, but it is noteworthy that DBN3a NS5A also showed a more diffuse cytoplasmic localization. This cell line will facilitate a more detailed analysis of the sub-cellular distribution of DBN3a NS5A in the context of the SGR, in comparison to both DBN3a_cc_-infected cells and other GTs (e.g. JFH-1).

**Fig. 4.**
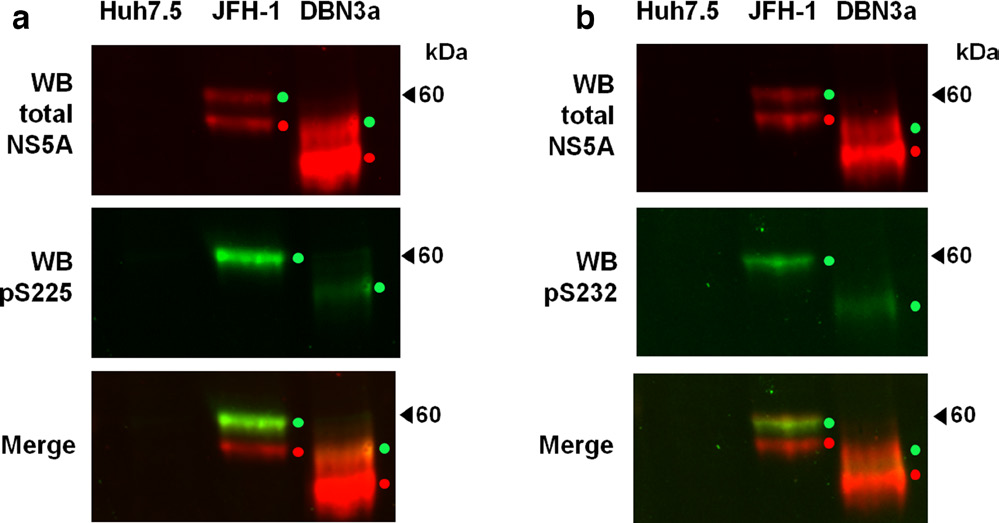
Phosphorylation of DBN3a NS5A. Lysates of Huh7 or Huh7.5 cells stably harbouring either SGR-feo-JFH-1 or SGR-neo-DBN3a were probed using a sheep polyclonal antiserum to detect total NS5A (red), together with phospho-specific antibodies to pS225 (a) or pS232 (b) (green signal). NS5A species are identified by either red dots (basally phosphorylated) or green dots (hyper-phosphorylated). Naïve Huh7.5 cells were included as a control.

We then used Western blotting to investigate the apparent molecular weight and potential for phosphorylation of DBN3a NS5A. In comparison to JFH-1, DBN3a NS5A was smaller (Fig. 4a, b, top panels), likely due to the presence of an 18 amino acid insertion near the C-terminus of JFH-1 NS5A [21]. Lysates from SGR-neo-JFH-1-harbouring cells showed the expected doublet representing basally and hyper-phosphorylated forms of NS5A at an equal intensity. We [18], and others [22], had previously shown that S52 only exhibited a single NS5A species [18]; this was the case both for the culture-adapted S52 [9], which contained the S2204I substitution, but also for wild-type S52, which maintained the phosphorylatable S2204 residue. This was perplexing, as S2204I was previously shown to abrogate hyper-phosphorylation in genotype 1b [23]. Unlike S52, DBN3a did not contain the S2204I substitution [which corresponds to S232 within the serine-rich low-complexity sequence I (LCSI)] and should therefore be able to undergo hyper-phosphorylation. Interestingly, DBN3a NS5A did exhibit the expected two bands corresponding to basally and hyper-phosphorylated species, however, compared to JFH-1, the ratio of hyper : basally phosphorylated species was much lower. To investigate this further, we used antisera specific for NS5A phosphorylated at either S225 [16] or S232 [17]. These antisera had been shown to detect hyper-phosphorylated NS5A only, consistent with hierarchical phosphorylation of serine residues within LCSI. As shown in Fig. 4a, b, DBN3a NS5A was reactive with both antisera, demonstrating the presence of both pS225 and pS232. We conclude that DBN3a NS5A is hyper-phosphorylated within LCSI, resulting in a change in apparent mobility, as seen for JFH-1, but that the proportion of DBN3a NS5A that is hyper-phosphorylated is lower than for JFH-1.

### Use of the DBN3a SGRs for analysis of DAA resistance

**Fig. 5.**
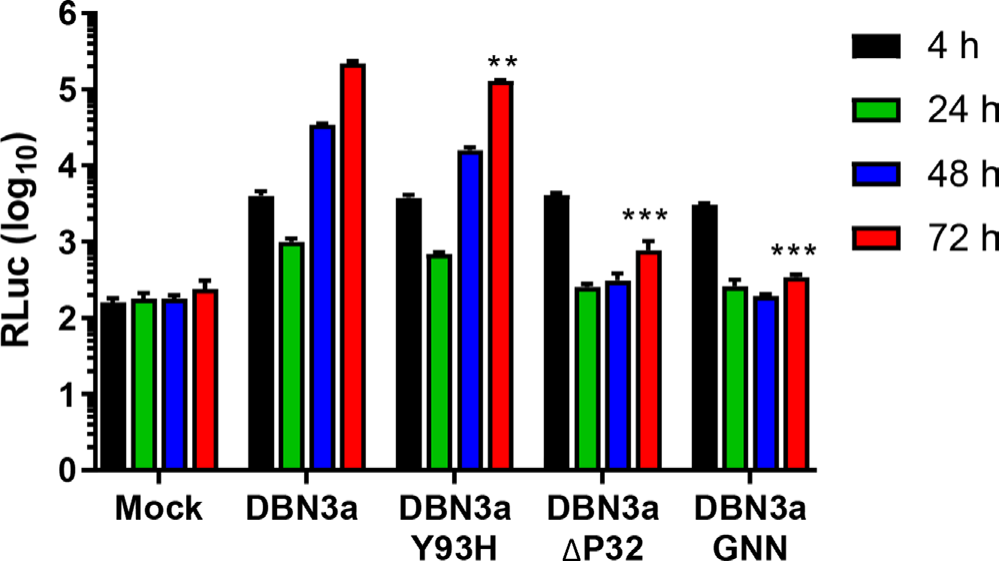
Replication of SGR-luc-DBN3a with resistance-associated substitutions (RAS). Huh7.5 cells were electroporated with *in vitro*-transcribed SGR-luc-DBN3a RNA with either the RAS Y93H or an in-frame deletion of proline 32 (ΔP32). Luciferase levels were measured at the indicated times and compared to mock, original and NS5B-GNN controls. *n*=3, error bars represent sem. **P <0.01, ***P <0.001 for 72 h p.e. values compared to original.

We proceeded to use the SGR-luc-DBN3a construct to evaluate the effect of DAAs on DBN3a genome replication. To do this we first generated two derivatives in which previously characterized resistance-associated substitutions (RASs) were engineered into NS5A. The first of these was Y93H, shown to be the predominant RAS in most genotypes, including GT3 [24, 25]. Another RAS that confers potent resistance to DAAs and is associated with treatment failure is a deletion of proline 32 (ΔP32). However, although this RAS has been reported in GT1 [26, 27], it has not been detected in patients with GT3 infection. As *in vitro* analysis of ΔP32 in the context of a chimeric GT2b/2a (JFH-1) virus demonstrated that it was unable to propagate [28], we reasoned that this might also be the case for this RAS in GT3. We therefore tested this using the SGR system. As shown (Fig. 5), introduction of Y93H showed a small but significant decrease in replication, reflecting data previously published for this mutation when introduced into both JFH-1 virus and replicons [29]. Introducing ΔP32 into the DBN3a replicon exhibited a significant impairment of replication, although replication was still occurring, as can be seen by the small increase in luciferase production at 72 h. Due to the impact of ΔP32 on replication, this mutant was not carried forward for DAA sensitivity assays.

**Fig. 6.**
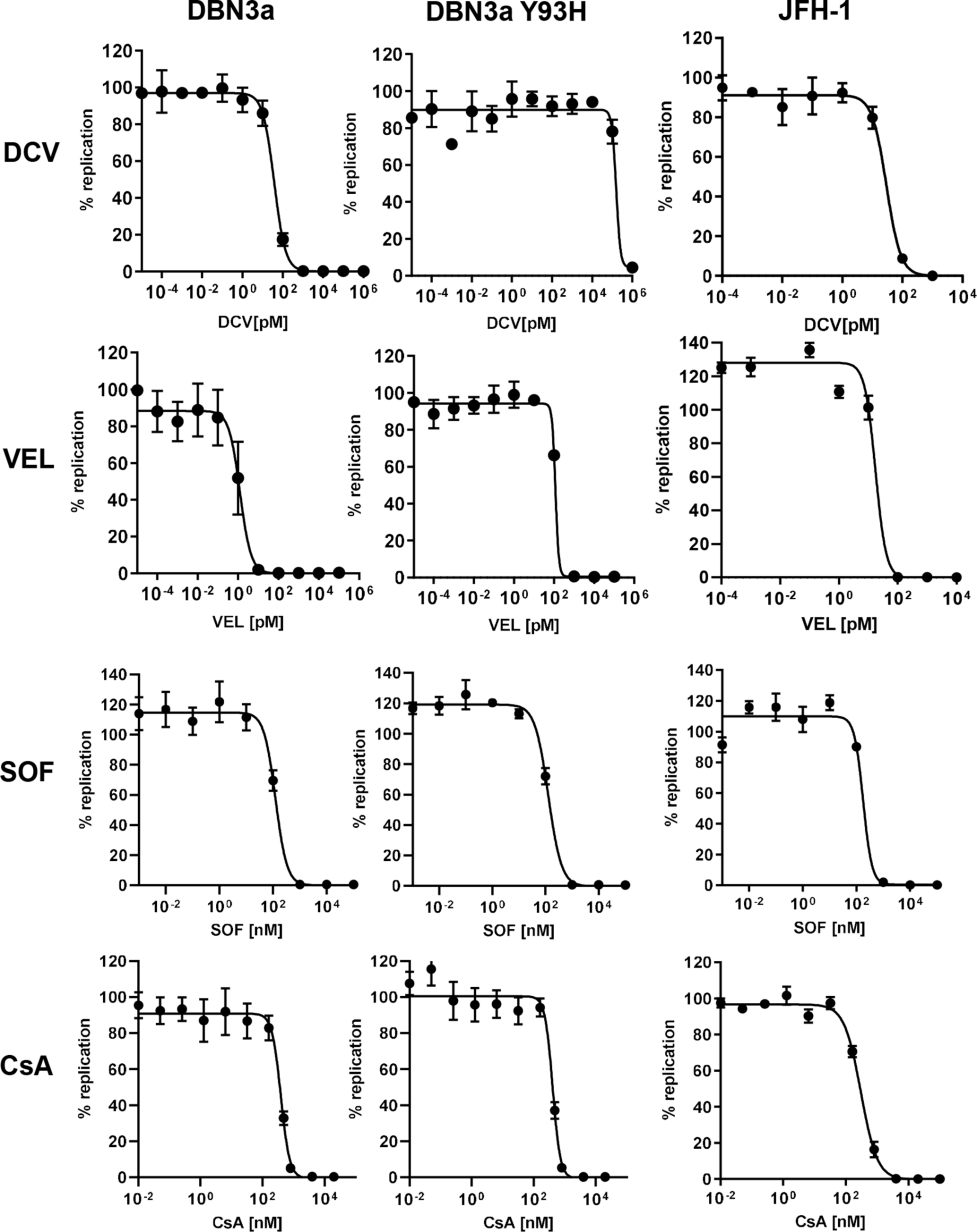
Response of SGR-luc-DBN3a to antiviral chemotherapeutics. Huh7.5 cells were electroporated with *in vitro*-transcribed SGR-luc-JFH-1 or SGR-luc-DBN3a (original or Y93H) RNA. Compounds were added at the indicated concentrations. Luciferase levels were measured at 72 h p.e. and the effective concentration 50 % (EC_50_) values were calculated using GraphPad Prism. *n*=3, error bars represent sem. DCV, daclatasvir; VEL, velpatasvir; SOF, sofosbuvir; CsA, cyclosporin A.

SGR-luc-DBN3a and the Y93H variant were used to ascertain the sensitivity of this GT3 strain to NS5A-specific DAAs that have been commonly used for treatment of GT3 infections (Fig. 6). SGR-luc-JFH-1 was used alongside these as a comparison. Velpatasvir (VEL) has largely superseded DCV as one of the NS5A DAAs of choice for treatment of GT3 infections [in combination with an NS5B inhibitor sofosbuvir (SOF)]. Therefore, as expected, the original SGR-luc-DBN3a was more sensitive to VEL than DCV (EC_50_ 1.3 pM vs 37 pM; Table 1). The Y93H RAS resulted in the acquisition of a high level of resistance to both compounds – for DCV a ~4000-fold increase in resistance (EC_50_ 156 nM) was observed, and for VEL a 90-fold increase (EC_50_ 118 pM) was observed. To confirm that this resistance was specific to the NS5A DAAs, we also evaluated the responses of the original and Y93H SGR to the NS5B DAA SOF and cyclosporin A (CsA, an inhibitor of the cellular peptidyl prolyl isomerase cyclophilin family – previously shown to be required for HCV genome replication). Y93H had no effect on the EC_50_ for either of these compounds. In comparison to JFH-1, the original DBN3a was 10-fold more sensitive to VEL but had similar EC_50_ values for the other inhibitors. Of note, the SGR-luc-DBN3a was more sensitive to all DAAs in comparison to the infectious clone (DBN3a_cc_). The latter exhibited an EC_50_ of 885 nM (SOF), 345 pM (DCV) and 24 pM (VEL) [12].

**Table 1.** EC_50_ values for antiviral compounds

	SGR-luc-DBN3a	SGR-luc-DBN3a Y93H	SGR-luc-JFH-1	DBN3a_cc_*
Cyclosporin A	389 nM	408 nM	300 nM	nd
Sofosbuvir	124 nM	127 nM	177 nM	885 nM
Daclatasvir	37 pM	156 nM	20 pM	345 pM
Velpatasvir	1.3 pM	118 pM	17 pM	24 pM

* Taken from [12]. nd, not determined.

## Discussion

The objective of this work was to generate a robust sub-genomic replicon system for GT3 HCV that would facilitate studies of both viral genome replication and DAA resistance, without the need for the biosafety level 3 (BSL3) containment facilities required in many countries, including the UK. Although two GT3a SGR constructs were developed previously [8, 9], these showed very limited replication capacity and replication of these SGRs could only be detected in stable SGR-harbouring Huh7 cells by qRT-PCR. Detection of genome replication using derivatives of S52 with a luciferase reporter was subsequently demonstrated [13], but the levels of luciferase declined by 48 h p.e. and only returned to input values by 96 h p.e. In contrast, the DBN3a-derived SGR containing a low-CpG/UpA-luciferase reporter that we developed in this study exhibited an approximately 100-fold increase in luciferase values by 48 h p.e. (Fig. 2a). This was almost as efficient as the gold standard GT2a JFH-1 SGR. Although SGR-luc-DBN3a replicated efficiently, we did observe a significant lag when compared to JFH-1 (Fig. 2a). This was not dependent on the reporter, as it was also observed with an eGFP reporter assayed kinetically using the IncuCyte ZOOM (Fig. 2b). An advantage of this system is the ability to monitor the relative rates of replication – this also showed that, after the lag, DBN3a replication was noticeably slower than JFH-1. However, it should be noted that the SGR assay is an indirect measurement of genome replication as the read-out directly measures reporter translation. It will be of interest to investigate the reasons underpinning this lag, as it may provide insights into potential biological differences between GT3 and other GTs. The SGR constructs generated here will be of great utility in such studies.

Importantly, our studies took advantage of cell culture-adaptive NS3-NS5B substitutions that previously permitted the development of an efficient infectious culture system of DBN3a [12]. This is a novel approach, since most previously developed SGRs depended on mutations selected for enhanced HCV RNA replication [30, 31], mutations that in some cases are detrimental for HCV infectivity [32]. Since cell culture infectious systems depending on adaptive replicase substitutions have been developed for several HCV genotypes [33], the approach used here could potentially benefit the development of robust SGRs for other HCV strains and genotypes.

One application of the SGR system generated here is to facilitate functional studies of NS5A. As proof of principle, we addressed the phosphorylation status of DBN3a NS5A. In this regard the potential function of NS5A phosphorylation remains enigmatic, although a number of recent studies have identified both sites of phosphorylation and the phenotypes of mutations at these sites [20, 34, 35]. These studies have focused on the GT2a isolate, JFH-1, and there is a dearth of information regarding NS5A phosphorylation in other GTs [18]. We show here that DBN3a NS5A exhibits both hyper- and basally phosphorylated forms by Western blot (Fig. 4). However, compared to JFH-1, there was a lower proportion of the hyper-phosphorylated form, which was reactive with antibodies to both phospho-S225 and phospho-S232, consistent with the conservation of the sequence within LCSI. During this experiment we noted that DBN3a NS5A species were less clearly defined by Western blot compared to JFH-1, possibly indicative of some other post-translational modifications, which should be addressed in future studies, including mass spectrometry analysis of NS5A. It will also be of interest to determine whether DBN3a NS5A mediates an increase in phosphatidylinositol 4-phosphate (PI4P) lipids in SGR-harbouring cells. In the context of the S52 GT3a isolate, the acquisition of culture-adaptive mutations (including S2204I) resulted in a loss of PI4P induction [22], which did not appear to correlate with the presence or absence of the hyper-phosphorylated NS5A species.

GT3 infections are responsible for 30 % of HCV infections globally and, importantly with regard to future reduction in HCV burden, GT3 is associated far more often with failure of treatment due to RAS compared to other GTs [4]. The DBN3a SGR described here will be of utility in elucidating the molecular basis for the high level of DAA resistance of GT3. As proof of principle, we engineered two well-characterized RASs (ΔP32 and Y93H) into SGR-luc-DBN3a. Although ΔP32 has been observed in GT1 and indeed is a potent driver of DAA resistance [26, 27], it has not yet been found in any clinical GT3 isolates. The reason for this could be related to our observation that this mutation caused a significant decrease in the replicative fitness of SGR-luc-DBN3a (Fig. 5). This again may point to a critical functional role of P32 that is GT-specific and allude to a biological difference between GT3 NS5A and other GTs.

In contrast, Y93H only displayed a modest, but significant, effect on SGR-luc-DBN3a replication and, as expected, resulted in a high level resistance to the NS5A-targeted DAAs, DCV and VEL (4200- and 90-fold, respectively). Of note, this study confirmed the pan-genotypic activity of VEL; even Y93H DBN3a replication was effectively inhibited at picomolar levels, consistent with clinically attainable concentrations [7]. In contrast, DCV, which was previously used in the treatment of HCV, would most likely not inhibit Y93H DBN3a clinically, as concentrations 1000-fold higher than for VEL were needed *in vitro* (Fig. 6). As mentioned, the DBN3a SGR was more sensitive to DAAs than the corresponding infectious clone, particularly for the NS5A-targeting DAAs (10–20-fold). The reasons for this difference are unclear, as the opposite effect was originally observed for GT2a (JFH-1) [36]. It may be indicative of functional and structural differences between the NS5A protein of the two GTs and merits further investigation. It is also noteworthy that the assay systems differ for infectious virus and SGRs, with the former measuring the number of infected cells, rather than luciferase activity.

In summary, we have shown improved tools for the study of GT3, with the modernization of existing systems to allow more detailed scrutiny of replication. The use of HCV replicase with infectious cell culture-adaptive mutations could pave the way for the development of robust SGRs of other HCV genotypes. We report key differences in GT2 and GT3 replication, with a delay in GT3 reporter expression and differences in the phosphorylation and DAA sensitivity of NS5A. The robust SGRs developed here will be of utility in dissecting both the high level of DAA resistance and differences in clinical outcomes observed with GT3.
